# Cognitive Impairment and Disability in Older Japanese Adults

**DOI:** 10.1371/journal.pone.0158720

**Published:** 2016-07-14

**Authors:** Hiroyuki Shimada, Hyuma Makizako, Takehiko Doi, Kota Tsutsumimoto, Sangyoon Lee, Takao Suzuki

**Affiliations:** 1 Department of Preventive Gerontology, Center for Gerontology and Social Science, National Center for Geriatrics and Gerontology, Obu, Japan; 2 National Center for Geriatrics and Gerontology, Obu, Japan; 3 Department of Gerontology, J.F. Oberlin University Graduate School, Tokyo, Japan; University of Granada, SPAIN

## Abstract

The prevalence of disability is increasing due to an expanding aging population and an increasing incidence of chronic health problems. Cognitive impairment may predict the development of disability in older adults. Therefore, we examined the association of mild cognitive impairment (MCI) and/or general cognitive impairment (GCI, defined as a Mini Mental State Examination [MMSE] score of 20–23) with the development of disability in a cohort of Japanese community-dwelling older adults. A total of 4290 participants (aged ≥65 years) enrolled in the Obu Study of Health Promotion for the Elderly were classified according to the presence and degree of cognitive impairment as follows: cognitively healthy, GCI, MCI single domain (MCIs), MCIs with GCI, MCI multiple domain (MCIm), and MCIm with GCI. MMSE scores, risk factors for dementia, and incidences of new disability were recorded. After an average of 29.5 months, 205 participants (4.8%) experienced a new onset of disability. All subtypes of cognitive impairment showed significant relationships with disability except for GCI alone. The following hazard ratios (HRs) were determined: MCIs (HR, 2.04; 95% CI, 1.39–3.00), MCIs with GCI (HR, 2.10; 95% CI, 1.21–3.62), MCIm (HR, 2.32; 95% CI, 1.39–3.85), and MCIm with GCI (HR, 4.23; 95% CI, 2.73–6.57). These results indicate that cognitive impairment may be related to an increased risk for the development of disability. Healthcare providers should implement global cognitive assessments to identify MCI and GCI and consider preventive interventions for disability, especially in older persons.

## Introduction

Approximately 15% of world’s population are estimated to have some form of disability. This rate is increasing due to an expanding aging population and increases in the prevalence of chronic health conditions such as dementia [1]. Cognitive impairment is one factor that can be associated with disability in older adults [2].

Mild cognitive impairment (MCI) is associated with increased risk of developing Alzheimer’s disease [3]. The National Institute on Aging and Alzheimer’s Association workgroup developed the following criteria for to describe the symptomatic pre-dementia (MCI) phase of Alzheimer’s disease: i) concern regarding cognitive changes, ii) impairment in one or more cognitive domains, iii) preservation of independence in functional abilities, and iv) no dementia [4]. The MCI diagnostic criteria of the Alzheimer’s Disease Neuroimaging Initiative include a Mini Mental State Examination (MMSE) [5] score of 24–30, subjective memory loss and objective memory loss as measured by education-adjusted scores on the Wechsler Memory Scale Logical Memory-II [6], a clinical dementia rating of 0.5, absence of significant levels of impairment in other cognitive domains, preserved activities of daily living (ADL), and no dementia. When the Alzheimer’s Disease Neuroimaging Initiative criteria are used to determine MCI, the subjects are divided as follows: cognitively healthy, MCI, general cognitive impairment (GCI; defined by MMSE score), and dementia.

The MMSE is useful for examining patients with an increased risk for developing dementia [7], but it demonstrated low specificity when the cutoff point was adjusted to ensure sensitivity [6]. Thus, use of the MMSE in isolation has a limited ability to discriminate between demented and non-demented patients in a general population. We therefore developed the National Center for Geriatrics and Gerontology-Functional Assessment Tool (NCGG-FAT), which consists of multidimensional cognitive tasks that assess memory, attention, executive function, processing speed, and visuospatial skill, in order to better detect MCI in the general population [7,8]. In a previous national study, we used the NCGG-FAT and MMSE to distinguish subjects with MCI from those with GCI. MCI in elderly patients with cognitive impairment of multiple domains was previously associated with disability onset in community-dwelling older adults in Japan [8]. However, it is unknown whether combined or separate statuses of MCI and GCI affect the incidence of disability with increasing age. This study therefore examined whether disability incidence is associated with MCI, GCI, or a combined status in community-dwelling older adults. Moreover, we performed stratified analyses to examine the relationship between cognitive impairment and disability incidence in different subgroups defined by sex, age, and depressive symptoms, as these factors may have confounded the observation of ADL limitations [9, 10]. We hypothesized that combined status of MCI and GCI is better predictor of future disability than either individual cognitive impairment status.

## Methods

### Study Population

This prospective study included 5104 community-dwelling older adults (≥65 years) who were enrolled in the Obu Study of Health Promotion for the Elderly (OSHPE) [11] between August of 2011 and February of 2012. The inclusion criteria were: age ≥65 at the time of examination, Obu residency, and no previous participation in other studies. The exclusion criteria were: need for support or care as certified by the Japanese public long-term care insurance (LTCI) system, disability in basic ADL, and inability to undergo performance-based assessments [11]. All participants underwent a baseline OSHPE assessment including an interview and evaluations of physical and cognitive function. Participants were followed monthly and monitored for LTCI certification for at least two years. Subsequent exclusions were the development of Parkinson’s disease, stroke, depression, dementia, an MMSE score <20, or disability based on the LTCI system at baseline. Participants who died or moved to another city during the follow-up period were also excluded. Of 5104 participants who completed the baseline OSHPE assessment, 1023 were excluded during follow-up. The remaining 4290 participants were included in the final analysis. Written informed consent was obtained from all participants prior to study inclusion, and the study protocol was approved by the Ethics Committee of the National Center for Geriatrics and Gerontology.

### Baseline Examination and Data Collection

#### Evaluation of cognitive function

We defined MCI [12–14] using the following criteria: i) objective cognitive impairment (indicated by an age- and education-adjusted score of at least 1.5 standard deviations below the reference threshold on tests commonly used for detailed neuropsychological assessment); ii) no evidence of functional dependency (e.g., no need for supervision or external assistance in performing ADL); and iii) exclusion by the clinical criteria for dementia. Screening for MCI included a standardized interview that collected sociodemographic and lifestyle data, medical history, and functional status data (basic ADL including eating, grooming, walking, stair climbing, and bathing habits), and cognitive testing using the MMSE [5] and NCGG-FAT [11, 15]. Based on cognitive testing results, MCI was further divided into single-domain or multiple-domain MCI. Individuals with 20–23 points on the MMSE and no clinical indications of dementia were considered to have GCI [16].

The NCGG-FAT consists of the following domains: memory (word list memory-I (immediate recognition); word list memory-II (delayed recall)), attention (an electronic tablet version of the Trail Making Test, TMT-part A), executive function (an electronic tablet version of the TMT-part B), and processing speed (an electronic tablet version of the Digit Symbol Substitution Test). Participants were given approximately 20 min to complete the battery. High test-retest reliability and moderate-to-high validity were confirmed in community-dwelling older adults for all NCGG-FAT components [15]. All tests had previously established standardized thresholds for defining cognitive impairment in the corresponding domain (a score less than 1.5 standard deviations below the age-specific mean) derived from a population-based OSHPE cohort of healthy older adults.

Finally, six groups were used to categorize presence and degree of cognitive impairment according to MCI status and MMSE score: 1) cognitively healthy with no MCI and no GCI; 2) GCI with an MMSE score of 20–23 points; 3) MCI single domain without GCI (MCIs), 4) MCIs with GCI, 5) MCI multiple domain without GCI (MCIm), and 6) MCIm with GCI. We did not distinguish between amnestic MCI and non-amnestic MCI in this study to avoid excessive classification.

#### Determination of disability

In the present research, participants were tracked monthly for new incidents of LTCI certification as recorded by the Japanese LTCI system, which is managed in each municipal government. The LTCI classifies a person as “Support Level 1 or 2” to indicate a need for assistance to support ADL or “Care Level 1 through 5” to indicate a need for continuous care [17]. In this study, disability was defined as an LTCI certification at any level. We defined disability onset as the point at which a participant was certified by the LTCI to require care.

#### Potential confounding factors of ADL

We selected two demographic variables, three physiological variables, four primary diseases or geriatric syndromes, and six psychosocial variables as possible confounding factors of ADL limitation (Table 1) [9, 10, 18]. The demographic variables included age and sex. The physiological variables “overweight” and “underweight” were determined using body mass index (BMI) measurement, and the cut-off points were 27.5 kg/m^2^ and 18.5 kg/m^2^, respectively [18]. Primary diseases and medical information were obtained via self-reporting and interview surveys. The following diseases were noted: heart disease, pulmonary disease, osteoarthritis, diabetes, and knee pain. Depressive symptoms were measured using the 15-item Geriatric Depression Scale (GDS) [19]. Instrumental activities and social roles were assessed using subscales of the Kihon-Checklist (with “yes” or “no” responses) [20].

**Table 1:** Participant Characteristics.

	Overall (n = 4290)	No MCI No GCI (n = 2944)	GCI (n = 301)	MCIs (n = 561)	MCIs with GCI (n = 162)	MCIm (n = 187)	MCIm with GCI (n = 135)	P
**Demographic variables**								
Age, years	71.8 ± 5.3	71.2 ± 5.0	72.7 ± 5.6	72.0 ± 5.3	74.8 ± 6.3	72.9 ± 5.8	75.4 ± 6.2	<.001
Sex, female	51.5	53.6	36.9	51.3	34.6	58.8	49.6	<.001
Physiological variables*								
Overweight, BMI 27.5 kg/m^2^, % yes	4.3	4.3	2	3.4	6.2	5.9	8.1	0.029
Underweight, BMI < 18.5 kg/m^2^, % yes	9	8.2	10	10.2	8.6	9.6	17	0.014
Knee pain, % yes	22.8	22.2	23.9	25.3	23.5	23	23	0.714
**Primary disease**								
Heart disease, % yes	15.5	15.2	16.9	17.3	11.7	16	16.3	0.558
Pulmonary disease, % yes	10.6	10.8	11	9.4	13.6	9.1	8.9	0.653
Osteoarthritis, % yes	13.7	13.2	13.3	17.5	13	13.4	9.6	0.095
Diabetes, % yes	13	12.8	11	13.9	12.3	17.1	13.3	0.477
**Psychological and social variables**								
MMSE	26.4 ± 2.5	27.3 ± 1.8	22.4 ± 0.9	26.6 ± 1.8	22.0 ± 1.0	26.1 ± 1.6	21.7 ± 1.0	<.001
Geriatric depression scale-15, score	2.7 ± 2.5	2.5 ± 2.4	2.6 ± 2.3	3.1 ± 2.6	3.5 ± 2.7	3.3 ± 2.5	3.8 ± 2.6	<.001
Going outdoors by bus and train, % no	8.7	7.6	7.3	10.9	9.9	13.9	17	<.001
Shopping for daily needs, % no	3.1	2.7	2.3	2.9	5.6	4.3	8.1	0.003
Visiting friends’ home,% no	12.6	11.2	12.6	15.3	16.7	16	23.7	<.001
Being called on for advice, % no	7.8	6.6	6.3	8.7	10.5	15	20.7	<.001

Data represent the mean ± standard deviation or percentage.

*The physiological variables “overweight” and “underweight” were determined using body mass index (BMI) measurement, and the cut-off points were 27.5 kg/m^2^ and 18.5 kg/m^2^, respectively [18].

### Statistical analyses

A one-way analysis of variance, Student’s *t*-test, and Pearson’s chi-square test were used to test differences in baseline characteristics among the six groups categorizing the presence and degree of cognitive impairment, and between participants with and without disability.

We calculated the cumulative incidence of disability during follow-up according to baseline cognitive impairment status and assessed inter-group differences using the Log-Rank test.

Cox proportional hazards regression models were used to analyze associations between cognitive impairment and the incidence of disability. Model 1 was adjusted for two demographic variables. Model 2 was adjusted for two demographic variables, three physiological variables, four primary diseases or geriatric syndromes, and six psychosocial variables as possible confounding factors. We estimated adjusted hazard ratios (HRs) and 95% confidence intervals (CIs) for the incidence of disability.

Stratified analyses were conducted to examine relationship between cognitive impairment and the incidence of disability in different subgroups defined by sex, age (<74 years or ≥75 years), and depressive symptoms (GDS score of 5/6) [21]. Adjusted HRs and 95% confidence intervals for the incidence of disability were also estimated in stratified analyses. All analyses were performed using IBM SPSS Statistics 20.0 (IBM Japan Tokyo). The level of statistical significance level was set at P<0.05.

## Results

A total of 4290 participants (mean age, 71.8±5.3 years; 51.5% female) were included in the present analysis. The numbers of participants diagnosed with GCI and MCI were 598 (13.9%) and 748 (17.4%), respectively. The prevalence of each subtype of cognitive impairment was as follows: 7.0% GCI, 13.1% MCIs, 3.8% MCIs with GCI, 4.4% MCIm, and 3.1% MCIm with GCI. During the follow-up period (average 29.5±3.9 months), 205 participants (4.8%) acquired LTCI certification.

Table 1 summarizes the possible confounding factors of ADL limitation for each subtype. All demographic, physiological, psychological, and social variables exhibited significant between-group differences, except for knee pain. Primary disease status was not statistically different among subtypes of cognitive impairment. Participants who developed disability were older, were more often women, were more often overweight, suffered from knee pain and heart disease, had a lower MMSE score and a higher GDS score, and reported lower instrumental and social activities than those who remained independent (Table 2).

**Table 2:** The incidence of disability according to possible confounding factors.

	Participants without disability (n = 4085)	Participants with disability (n = 205)	P
**Demographic variables**			
Age, years	71.4 ± 5.1	78.1 ± 6.2	<0.001
Sex, female	51	62	0.002
**Physiological variables**			
Overweight, BMI 27.5 kg/m^2^, % yes	4.1	7.8	0.011
Underweight, BMI < 18.5 kg/m^2^, % yes	8.8	12.7	0.055
Knee pain, % yes	22.4	31.2	0.003
**Primary disease**			
Heart disease, % yes	15.1	24.9	<0.001
Pulmonary disease, % yes	10.4	14.1	0.089
Osteoarthritis, % yes	13.5	17.6	0.096
Diabetes, % yes	12.8	16.6	0.116
**Psychological and social variables**			
Mini-mental state examination, score	26.5 ± 2.4	25.0 ± 2.7	<0.001
Geriatric depression scale-15, score	2.6 ± 2.5	3.7 ± 2.7	<0.001
Going outdoors by bus and train, % no	8.3	16.1	<0.001
Shopping of daily necessaries, % no	3	3.9	0.469
Visiting the homes of friends, % no	12.1	22.4	<0.001
Being called on for advice, % no	7.4	15.6	<0.001

**Fig 1:**
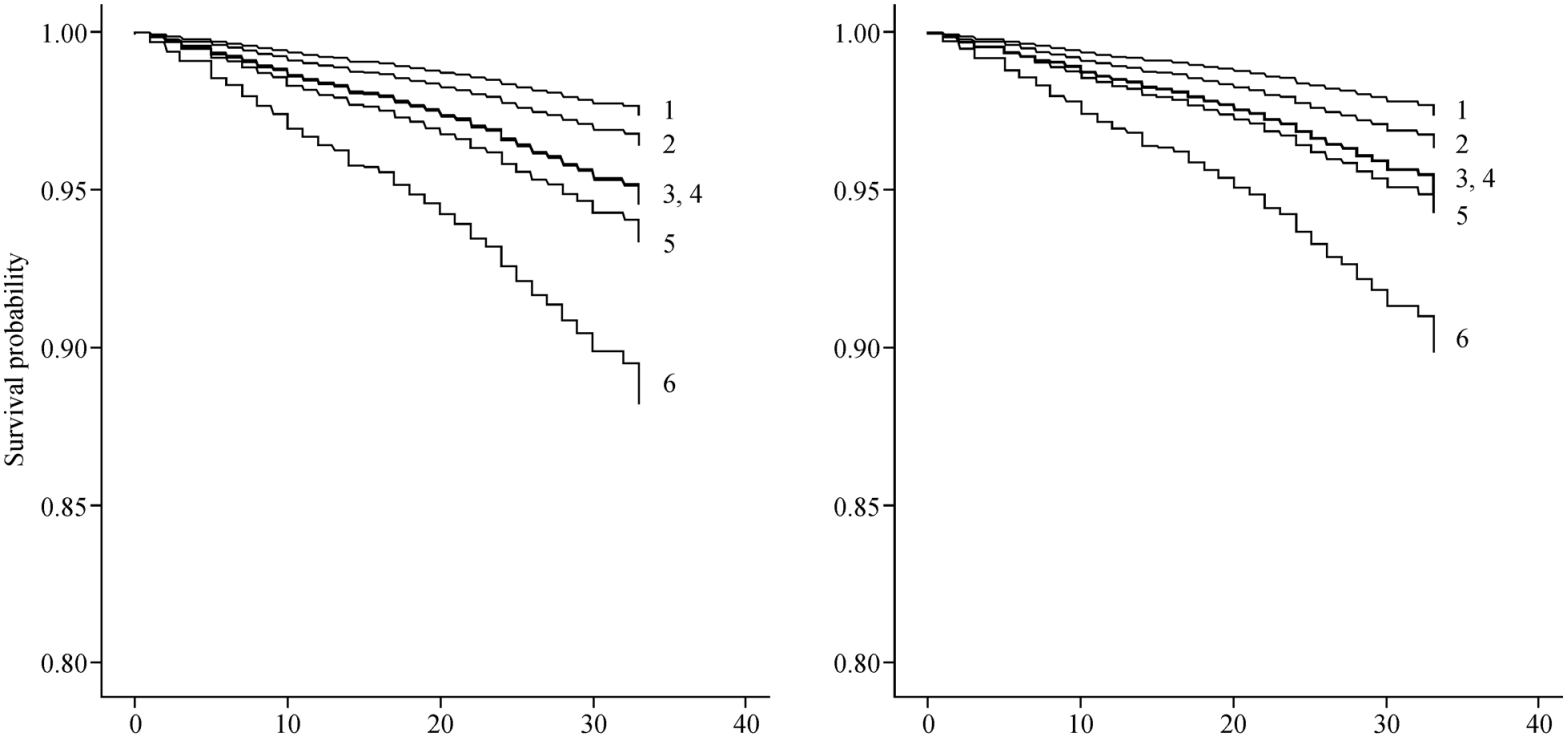
Estimated survival rates according to the incidence of disability and cognitive impairment status. The left panel shows survival curves according to the incidence of disability adjusted for age and sex, and the right panel shows the fully adjusted model. 1, non-cognitive impairment group; 2, non-mild cognitive impairment with general cognitive impairment group; 3, mild cognitive impairment single domain group; 4, mild cognitive impairment single domain with general cognitive impairment group; 5, mild cognitive impairment multiple domain group; 6, mild cognitive impairment multiple domain with general cognitive impairment group.

Next, Cox proportional hazards regression models were used to analyze associations between cognitive impairment and the incidence of disability (Table 3 and Fig 1). In Model 1, the following HRs were determined for each group: GCI (HR, 1.38; 95% CI, 0.79–2.39), MCIs (HR, 2.06; 95% CI, 1.40–3.02), MCIs with GCI (HR, 2.10; 95% CI, 1.22–3.61), MCIm (HR, 2.56; 95% CI, 1.56–4.19), and MCIm with GCI (HR, 4.65; 95% CI, 3.03–7.12). Age and sex were significantly associated with the incidence of disability. In the fully adjusted Model 2, all subtypes of cognitive impairment remained significant except the GCI group. The following HRs were determined for each group: MCIs (HR, 2.04; 95% CI, 1.39–3.00), MCIs with GCI (HR, 2.10; 95% CI, 1.21–3.62), MCIm (HR, 2.32; 95% CI, 1.39–3.85), and MCIm with GCI (HR, 4.23; 95% CI, 2.73–6.57). In Model 2, age, sex, and heart disease were significantly associated with the incidence of disability.

**Table 3:** Hazard ratios for the incidence of disability according to cognitive impairment status and confounding factors.

	Model 1		Model 2	
	Hazard ratio (95% CI)	P	Hazard ratio (95% CI)	P
Age, years	1.16 (1.14–1.18)	<0.001	1.15 (1.13–1.18)	<0.001
Sex, female/male	0.64 (0.48–0.85)	0.002	0.63 (0.47–0.85)	0.003
Overweight, BMI 27.5 kg/m^2^, % yes			1.24 (0.72–2.12)	0.435
Underweight, BMI < 18.5 kg/m^2^, % yes			1.49 (0.97–2.27)	0.068
Knee pain, % yes			1.18 (0.85–1.64)	0.325
Heart disease, % yes			1.41 (1.02–1.96)	0.04
Pulmonary disease, % yes			1.29 (0.87–1.91)	0.214
Osteoarthritis, % yes			0.82 (0.55–1.23)	0.333
Diabetes, % yes			1.31 (0.90–1.90)	0.159
Geriatric depression scale-15, score			1.04 (0.99–1.10)	0.137
Going outdoors by bus and train, % yes			1.42 (0.93–2.16)	0.105
Shopping of daily necessaries, % yes			0.51 (0.23–1.12)	0.094
Visiting the homes of friends, % yes			1.38 (0.94–2.04)	0.104
Being called on for advice, % yes			1.16 (0.75–1.81)	0.507
Cognitive impairment		<0.001		<0.001
Non-Cognitive impairment	1		1	
GCI	1.38 (0.79–2.39)	0.257	1.43 (0.82–2.49)	0.207
MCIs	2.06 (1.40–3.02)	<0.001	2.04 (1.39–3.00)	<0.001
MCIs with GCI	2.10 (1.22–3.61)	0.007	2.10 (1.21–3.62)	0.008
MCIm	2.56 (1.56–4.19)	<0.001	2.32 (1.39–3.85)	0.001
MCIm with GCI	4.65 (3.03–7.12)	<0.001	4.23 (2.73–6.57)	<0.001

Next, we conducted survival analyses using the Kaplan-Meier Log-Rank Tests. The results showed that the probability of disability incidence was significantly higher in participants with GCI (P = 0.047), MCIs (P<0.001), MCIs with GCI (P<0.001), MCIm (P<0.001), or MCIm with GCI (P<0.001) than in cognitively healthy control participants. The probability of disability incidence was significantly higher in participants who had MCIs with GCI (P = 0.047), MCIm (P = 0.018), or MCIm with GCI (P<0.001) than in those with GCI. The MCIm with GCI group showed a higher risk of disability incidence than the MCIs (P<0.001), MCIs with GCI (P = 0.001), and MCIm (P = 0.003) groups.

**Fig 2:**
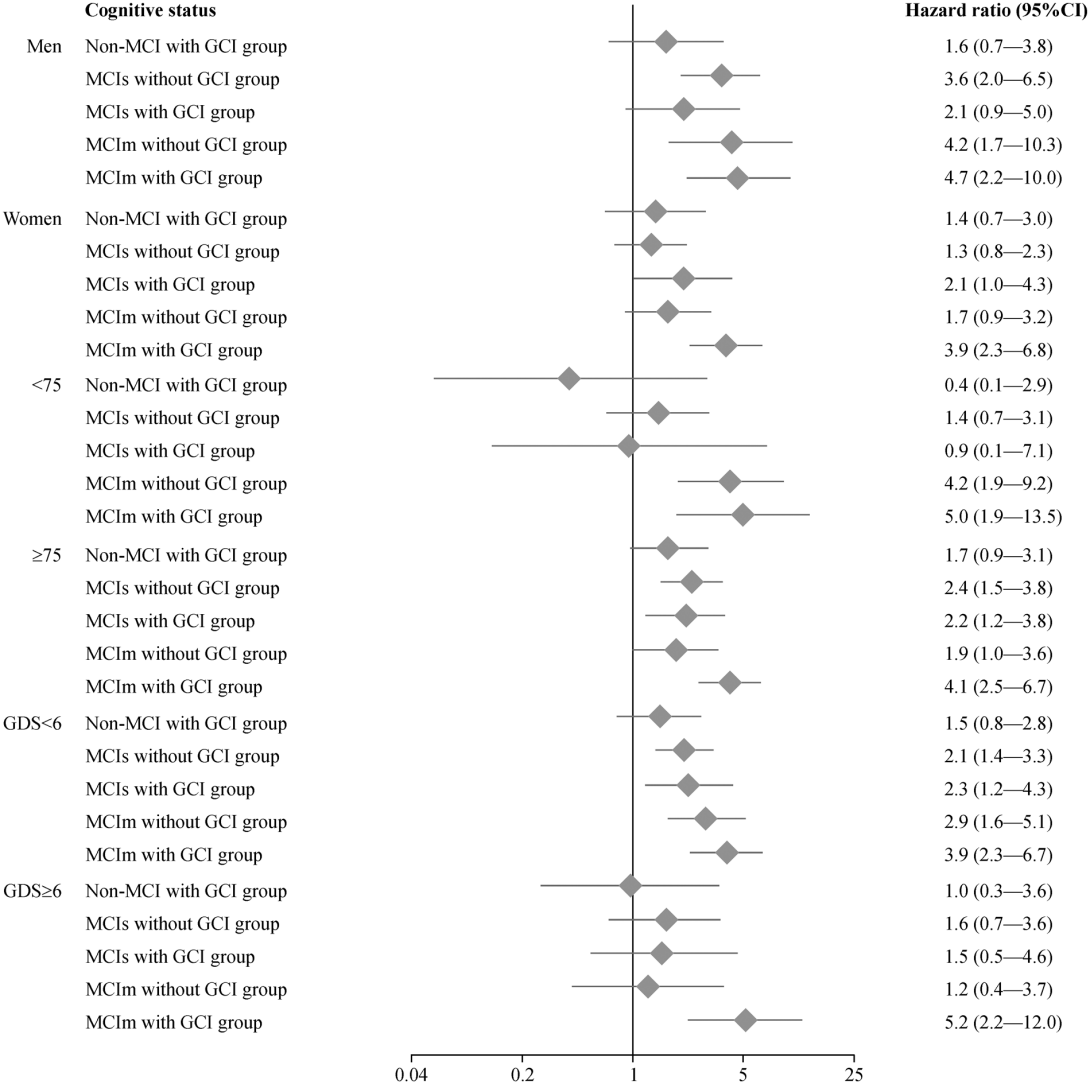
Hazard ratio estimates of relative disability risk in subgroups defined by sex, age, and depressive symptoms in stratified analyses.

Finally, we conducted stratified analyses that were divided by sex, age, and depressive symptoms (Fig 2). The MCIm with GCI group showed significantly higher HRs in all stratified subgroups than the cognitively healthy group. The following HRs were determined in subgroups of the MCIm with GCI group: males (HR, 4.7; 95% CI, 2.2–10.0), females (HR, 3.9; 95% CI, 2.3–6.8), participants aged <75 years (HR, 5.0; 95% CI, 1.9–13.5), participants aged ≥75 years (HR, 4.1; 95% CI, 2.5–6.7), participants with <6 points on the GDS (HR, 3.9; 95% CI, 2.3–6.7), and participants with ≥6 points on the GDS (HR, 5.2; 95% CI, 2.2–12.0).

## Discussion

In the present study, we concluded that a baseline diagnosis of MCI or MCI with GCI was significantly associated with the development of disability. This conclusion was valid even after controlling for confounding factors such as sociodemographic information and depression symptoms. In previous studies, poorer cognitive function has been identified as a significant predictor of functional decline even among older adults with cognitive function in the normal range [22]. Conversely, functional disability in ADL has also been proposed to contribute to cognitive decline [23]. Based on these previous studies, cognitive decline and functional disability are associated and may interact synergistically, explaining the observations of the present study.

Previous studies in other countries have addressed the prevalence of MCI and GCI in elderly populations [24–26]. In the Cardiovascular Health Study Cognition Study, the prevalence of MCI among 2,470 older adults was 18.8% [26]. In the Medical Research Council Trial, the prevalence of GCI among 15,051 community-dwelling older people was 18.3% [24]. In our cohort, the prevalence of MCI and GCI were 17.4% and 13.9%, respectively, which is consistent with these previous reports. However, to our knowledge, no study to date has clearly identified the prevalence of MCI with or without GCI in a large sample. In our study, the prevalence rate GCI without MCI was 7%. These results highlight the importance of multi-faced screening for the evaluation of dementia risk, and suggest that current methods evaluating only GCI or MCI may underestimate or overlook a target population requiring preventive intervention for dementia.

GCI and MCI are closely associated with dementia onset in older adults [27]. A meta-analysis to determine the rate of progression from MCI to dementia showed that the annual conversion rate was 5.2% (95% CI, 2.9–8.0%) in community studies [27]. In contrast, the annual conversion rate was only 0.43% in healthy controls. Previous studies have also reported that older adults with MCI show performance declines in ADL, particularly in complex and cognitively demanding activities [28, 29]. Although the relationship between MCI and disability is not fully understood, these previous findings taken together with our current data indicate that MCI is a plausible risk factor for the development of disability.

The MMSE is the best-studied brief screening tool for measuring general cognition in the context of dementia; the reported sensitivity and specificity of the MMSE range from 71–92% and 56–96%, respectively [30]. The MMSE is also useful for identifying disability [31]. However, a drawback of the MMSE as a general cognitive test is its varying accuracy across subjects of different ages, education levels, and ethnicities [32–34]. Previous studies have compensated for this limitation by changing the cut-off score based on age and education level [30, 35]. In the present study, we used <24 points as the cut-off for GCI; therefore, subjects at the extremes of age and education were likely to be assessed inaccurately. Given this limitation, evidence of GCI alone may not reliably predict the future incidence of disability.

Our cognitive impairment classification allowed us to identify several significant trends of disability incidence even after adjusting for potential confounding factors. In the fully adjusted model, the MCIm with GCI group showed a higher HR for disability than the GCI, MCIs, MCIs with GCI, and MCIm groups. These results suggest that healthcare providers should more carefully evaluate the extent of cognitive decline in order to identify the need for preventive interventions for disability, especially in older persons.

In our sample, log-rank tests showed significant differences in disability incidence between subtypes of the MCIm with GCI group. Furthermore, in stratified analyses of sex, age, and depressive symptoms as major possible confounding factors of ADL limitation [9, 10], all MCIm with GCI group analyses showed the highest HRs for disability incidence. In contrast, no significant HRs were observed in stratified analyses of the GCI group. These results suggest that the combined status of MCIm and GCI is a strong predictor of disability. Cognitive assessments of MCI and GCI in clinical and community settings may therefore have an important role in preventing disability in older adults.

The main strengths of this study include a large sample size, the comprehensive nature of our assessments, and a prospective design that can address causality between predisposing factors and disability onset. An important limitation of our study is that patients were recruited non-randomly from a single community. Given that a majority of the participants recruited from Obu were relatively healthy elderly persons with regular access to health care, our sample may have underestimated the prevalence of cognitive impairment in the general population. Second, we were unable to contact informants (e.g., family members) for the verification of medical records, lifestyle information, and asymptomatic aberrant behaviors. Third, our follow-up period was relatively short and therefore had a limited the ability to identify the pattern of disability incidence over time.

In summary, the results of this prospective cohort study indicate that cognitive impairment has a strong impact on the risk of developing disability. In particular, community-dwelling older adults with combined MCI and GCI have an increased risk of disability incidence.
